# Tracking longitudinal language network reorganisation using functional MRI connectivity fingerprints

**DOI:** 10.1016/j.nicl.2021.102689

**Published:** 2021-04-30

**Authors:** Natalie L. Voets, Oiwi Parker Jones, Claire Isaac, Rogier B. Mars, Puneet Plaha

**Affiliations:** a Wellcome Centre for Integrative Neuroimaging, University of Oxford, Oxford, UK; b Department of Neurosurgery, Oxford University Hospitals NHS Foundation Trust, Oxford, UK; c Russell Cairns Unit, John Radcliffe Hospital, Oxford University Hospitals NHS Foundation Trust, Oxford, UK; d Donders Institute for Brain, Cognition and Behaviour, Radboud University Nijmegen, Nijmegen, the Netherlands; e Nuffield Department of Surgical Sciences, University of Oxford, Oxford, UK

**Keywords:** fMRI, Neuroplasticity, Longitudinal, Glioma, Neurosurgery, Language, Tumor

## Abstract

Large individual differences in how brain networks respond to treatment hinder efforts to personalise treatment in neurological conditions. We used a brain network fingerprinting approach to longitudinally track re-organisation of complementary phonological and semantic language networks in 19 patients before and after brain-tumour surgery. Patient task fingerprints were individually compared to normal networks established in 17 healthy controls. Additionally, pre- and post-operative patient fingerprints were directly compared to assess longitudinal network adaptations. We found that task networks remained stable over time in healthy controls, whereas treatment induced reorganisation in 47.4% of patient fluency networks and 15.8% of semantic networks. How networks adapted after surgery was highly unique; a subset of patients (10%) showed ‘normalisation’ while others (21%) developed newly atypical networks after treatment. The strongest predictor of adaptation of the fluency network was the presence of clinically reported language symptoms. Our findings indicate a tight coupling between processes disrupting performance and neural network adaptation, the patterns of which appear to be both task- and individually-unique. We propose that connectivity fingerprinting offers potential as a clinical marker to track adaptation of specific functional networks across treatment interventions over time.

## Highlights

• FMRI connectivity fingerprints represent patient-unique language networks.
• Fingerprints can be statistically tested to detect reorganisation in individuals.
• Connectivity fingerprints track surgery-related adaptations in individual patients.
• Network-level changes appear related to presence of language symptoms.

## 1 Introduction

Selective language impairments arise from damage to critical brain structures (Hope et al., 2013) within wide-scale networks supporting speech, comprehension and reading (Dick et al., 2014). However, even within specific language domains such as word generation, patients with comparable brain injuries display high variability in the magnitude of both their deficits and recoveries. This variability may in part reflect degeneracy within language networks (Friston and Price, 2011) but may also arise from individual differences in how brain networks adapt in the context of disease (Berl et al., 2014).

The ability to visualise individual differences in how functional networks are organised in the brain is directly relevant in clinical settings. For example, neurosurgical conditions such as drug-resistant epilepsy (Balter et al., 2016) and brain tumours (Cargnelutti et al., 2020) can induce reorganisation of language (among other) networks. Establishing the contribution of brain structures to language in the individual patient is important to minimize accidental damage that might induce profound language deficits (Bookheimer, 2007). Complexity arises from evidence that, at least after stroke (Saur et al., 2006) and surgery (Gil Robles et al., 2008, Helmstaedter et al., 2006) language networks may change dynamically over time.

Treatment interventions, such as rehabilitative therapies and drug interventions, may require a multi-stage approach to adapt treatments according to tolerance and response. For example, in the context of brain tumours, multiple surgeries may be needed because eloquent structures prevent complete removal in one go, or when a tumour grows back. Optimal treatment planning therefore requires an understanding not only of how language networks are represented in a patient’s brain at a given moment, but also, importantly, how they evolve over time (Duffau, 2014, Gil Robles et al., 2008, Sarubbo et al., 2012, Southwell et al., 2016). As a first step toward this goal, we recently evaluated a functional MRI (fMRI) ‘fingerprinting’ approach, able to visualise and statistically measure differences in functional connectivity among regions involved in word generation in untreated brain tumour patients (Voets et al., 2019). This was an important proof of principle, because classic population-based studies do not explore the huge inter-individual variations that underlie extreme differences in disease and treatment outcomes. Using this approach, we showed that tumours in the language-dominant hemisphere frequently disrupt the speech-related network in individually-unique ways. However, in order for this fingerprinting technique to have further potential to inform clinical treatment, it should not only detect brain network differences at one time-point but, furthermore, should also show sensitivity to track how networks evolve in individual patients, for example across an intervention.

Here, we therefore set out to further establish if fingerprint connectivity patterns a) dissociate adaptions within language networks connected to phonological and semantic tasks in individual patients, and b) detect pre- to post-operative network changes resulting from surgical intervention at the *single-subject level*. We conducted a longitudinal study to track the impact of surgical removal of a primary brain tumour (glioma) on the organisation of individual patient fingerprints. Patients underwent fMRI scans once before and once after surgery, and were compared to age- and gender-matched healthy controls, who were also scanned twice. To explore fingerprint specificity, we compared the networks associated with two fMRI tasks probing dissociable aspects of language: a letter fluency task representing phonological processing, and the Pyramids and Palm Trees Task (PPTT) representing semantic processing. To determine the relationship between fingerprints and performance, we evaluated whether the presence of (pre or post-surgical) clinical language symptoms impacted on network organisation.

## 2 Materials and methods

### 2.1 Participants

**Fig. 1:**
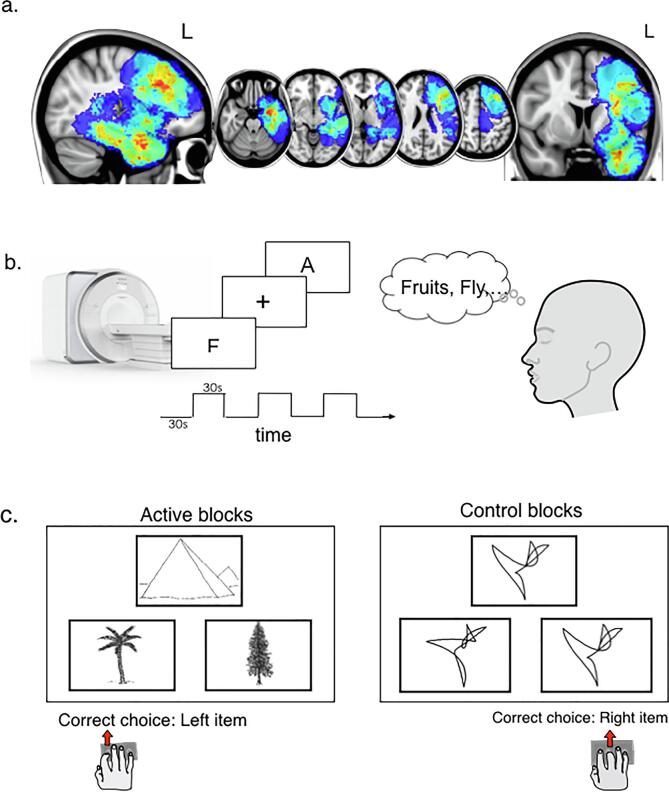
FMRI word generation and semantic association tasks *Legend*. a. Overlap heat maps of tumour locations in 19 patients radiologically diagnosed with a glioma in the language-dominant hemisphere. The language-dominant hemisphere was the left hemisphere in 18 right-handed patients and was the right hemisphere in 1 left-handed patient who presented with seizures affecting speech; the data for this patient were hemisphere-swapped in order to standardise the language-affected hemisphere to be the left hemisphere. (b) Phonological processing (word generation) was assessed using a block-design silent letter fluency task. Participants silently generated words beginning with the presented letter, or looked at a fixation cross for a task duration of 04min12s. (c) Semantic association was assessed using picture items from the Pyramids and Palm Trees Test, which were contrasted with non-nameable geometric line designs to control for aspects of the task (visual processing, decision making, response selection) not specific to semantic processing, and blocks of resting fixation. Task duration was 05min24s.

Adult patients with a radiological diagnosis of a solitary glioma involving the frontal or temporal lobe (Table 1, Fig. 1a) were prospectively recruited through the Oxford University Hospitals NHS Foundation Trust neuro-oncology surgery service. Exclusion criteria included: contraindications to MRI, cancer elsewhere in the body, and prior surgery other than biopsy. Twenty English-speaking patients with a radiologically diagnosed language-dominant hemisphere tumour underwent pre-operative fMRI. In one patient, post-operative histological analysis confirmed a focal cortical dysplasia. This patient was therefore excluded from the longitudinal assessment, leaving 19 patients with a confirmed glioma available for analysis (mean age 39.9 ± 13.9 years, range 22–70, 11 women). Patients in this series were operated between July 2014 and July 2018. All but 4 surgeries were performed awake. Patients were scanned as close as possible before surgery, and again after surgery. The post-surgical scan was scheduled just before starting radiotherapy for high grade gliomas (mean: 6.1 weeks post-operatively), or approximately 6 months (mean: 27.6 weeks) after surgery for patients not undergoing adjuvant treatment. Resections were radiological defined to be gross total (n = 4), near total (>90% resection, n = 10) and subtotal (<90% resected, n = 5).

**Table 1:** Patient clinical characteristics.

Clinical variable	Number of patients
Sex (Male/Female)	08/11
Handedness (Right/Left/ambidextrous)	17/1/1
Tumour location	
Temporal lobe	9
Frontal lobe	9
Insula	1
Pathology	
Low grade glioma	10
Oligodendroglioma (WHO II)	3
Astrocytoma (WHO II)	7
High grade glioma	9
Anaplastic Astrocytoma (WHO III)	5
Anaplastic Oligodendroglioma (WHO III)	1
Glioblastoma (WHO IV)	3
IDH status	
Wild-type	4
Mutated	15

WHO: World Health Organisation (grading system). IDH: Isocitrate dehydrogenase (gene mutations).

Controls were 17 right-handed healthy fluent-English speaking volunteers (mean age 36.6 ± 11.93 years, range: 26–68, 9 women), age-matched to the patient group (t(34) = 0.76, p = 0.45). Controls underwent two scans, timed to match the patient group’s average interval (mean 19.7 vs 18.1 weeks, t(34) = 0.38, p = 0.71). Controls were excluded if they had a previous or current neurological or psychiatric condition.

### 2.2 Ethics

The study was approved by the Oxford-B Research Ethics Committee and conducted in accordance with the principles of the Declaration of Helsinki. All participants gave informed written consent to take part.

### 2.3 Language tasks

FMRI data were acquired during two visually-cued language tasks (Fig. 1b,c). During word generation, probing phonological aspects of language, participants silently produced words beginning with a letter (F, A, S, M) for 30 s each, alternated with 30 s of resting fixation. Semantic association was probed using an adaptation of the Pyramids and Palm Trees Test (PPTT) (Howard and Patterson, 1992). This task was chosen as a step towards aligning fMRI assessments with intra-operative monitoring during awake glioma surgeries, performed for the majority of language dominant hemisphere tumours in our centre. During semantic task trials, participants were presented with three pictures, including a target (e.g. a pyramid), and two choice items (e.g. a pine and a palm tree). Participants selected which choice item was most closely related to the target. Control trials required visual matching of un-nameable drawings modified from Slotnick & Schacter (Slotnick and Schacter, 2004). Blocks lasted 16 s each, including 4 trial items presented for 4 s each. For both tasks, the order of blocks and the order of trials within blocks was held constant within and between participants. Tasks were practiced out loud before each scan.

Fluency performance was available before and after surgery in 15/19 patients, and recorded once in 14/17 controls. The total number of words generated (to letter F, A, S on the first visit and C, F, L on the second visit, one minute each) was summed and converted to established population-standardised normalised z-scores. Performance on the PPTT items (percent accuracy on semantic trials) was calculated from the raw response scores recorded during fMRI at each visit.

### 2.4 MRII

Blood-oxygen level dependent echo planar images were acquired on one of two 3 T MRI scanners at the University of Oxford. Scan sequence parameters are detailed in Supplementary Table 1. A high-resolution (1 mm^3^) T1-weighted scan was acquired for fMRI co-registration and manual measurement of tumour volumes.

### 2.5 FMRI pre-processing

Data were pre-processed using Melodic, part of the FMRIB Software Library (FSL, v5.0, www.fmrib.ox.ac.uk/fsl). Images were brain extracted, temporally filtered (90 s), motion corrected, corrected for geometric distortions using a separately computed fieldmap, spatially smoothed (5 mm), and aligned to the anatomical scan using tissue boundary-based registration (Greve and Fischl, 2009).

### 2.6 Regions of interest (ROIs)

One of the first challenges when testing for ‘change’ in language networks is to decide which brain regions to evaluate for evidence of longitudinal adaptation. Previous studies in stroke and chronic epilepsy patients indicate that when ‘reorganisation’ of language functions is observed, atypical language patterns involve, at least in gross terms, redistribution of activity across nodes in the existing network, including contralateral homologue regions (Mbwana et al., 2009, Turkeltaub et al., 2011). Therefore, comprehensive but specific language network ROIs were selected from literature reviews of regions involved in speech production and semantic processing. Neuroanatomical (Amunts et al., 2004, Petrides et al., 2012), functional imaging (Mechelli et al., 2007), connectivity (Anwander et al., 2007, Jakobsen et al., 2016) and neuromodulation (Duffau et al., 2005, Gough et al., 2005) studies all highlight functional subdivisions within the inferior frontal cortex. Among these, ventral pars opercularis (“Pop”) contributes preferentially to phonological (Lorca-Puls et al., 2017) and articulatory processing (Price, 2010). As previously described (Voets et al., 2019), we therefore selected left hemisphere Pop as the seed region for the word generation task connectivity fingerprint. Anatomical targets consisted of 16 regions selected to represent core speech network areas (Price, 2010). These included the middle frontal gyrus/inferior frontal sulcus, ventral premotor cortex, posterior superior temporal sulcus, anteroventral supramarginal gyrus, dorsal anterior cingulate cortex, caudate and putamen in both hemispheres, and the left anterior supplementary motor area and right pars opercularis (Supplementary Table 2).

For the semantic network, fMRI *meta*-analyses and studies in neurological populations identify a bilaterally-distributed network of regions supporting semantic access, retrieval and decision making (Binder et al., 2009). The single ‘most critical’ region in this network is not established. Left hemisphere inferior frontal region pars orbitalis (“Por”) is selectively involved in verbal semantic processing (Binder et al., 2009, Duffau et al., 2005, Gough et al., 2005, Mechelli et al., 2007). Por was therefore initially selected as the seed region for the semantic task fingerprint. Thirteen target regions were chosen from the literature based on their role in aspects of concept processing relevant to semantic knowledge (Binder et al., 2009): the anterior temporal pole, posterior fusiform gyrus, ventral angular gyrus, middle frontal gyrus/inferior frontal sulcus, pars triangularis and anterior cingulate cortex bilaterally (Supplementary Table 3). For each ‘seed’ and ‘target’ ROI, a 5 mm spherical mask was created on the Montreal Neurological Institute (MNI) template brain, centred on the literature coordinates. In order to examine the effect of varying ROIs, this analysis was repeated using the left temporal pole as the primary seed (see Supplemental Results).

### 2.7 Fingerprint analysis

Task functional connectivity maps were generated using seed-based correlation analysis (O'Reilly et al., 2010, Voets et al., 2019). First, we nonlinearly registered (accounting for lesion deformation by excluding voxels within tumour masks (pre-operatively) and surgical cavities (post-operatively) from the registration calculations) the Pop (for fluency) and Por (for PPTT) ‘seed’ masks to participants’ fMRI data. From the resulting subject-specific seed masks, we extracted the average fMRI signal time-series for each task, from each scan. Next, we calculated the correlation between the seed region (Pop/Por) and every voxel in the brain. The time-courses associated with white matter, head motion and cerebrospinal fluid were regressed out to minimize non-neuronal confounds (O'Reilly et al., 2010). Fischer z transforms were used to normalize the resulting *whole-brain* correlation maps (Supplementary Fig. S1).

To generate the *network-specific* fingerprints, we applied the pre-selected target ROIs to the whole-brain correlation maps, to extract the mean z-normalised correlation values with the relevant seed area (Pop/Por). These average correlation values were visualised as spider plots and used as input for statistical analyses.

Fingerprints were statistically analysed using permutation testing implemented in the MR Comparative Anatomy Toolbox for Matlab (MrCat, www.neuroecologylab.org) (Mars et al., 2013, Mars et al., 2016). Permutation testing (10,000 permutations) was conducted to evaluate the Manhattan Distance (MD) between healthy control group fingerprints over time, and for individual patient fingerprints, as described previously (Mars et al., 2013, Mars et al., 2016, Voets et al., 2019). Smaller MDs reflect greater similarity between fingerprints. Three sets of analyses were conducted, to:

#### 2.7.1 Assess temporal stability in controls

Before evaluating surgery-related network changes in patients, we tested if fingerprints varied over time in controls. Using permutation testing, we plotted the distribution of the MD statistic between the visit 1 and visit 2 fingerprints for both tasks, separately. We established a one-tailed significance criterion, representing the value at which the between-visit difference in fingerprints is larger than would be expected by chance. Next, we established the actual statistic measured from the data. If p < 0.05, the fingerprints were deemed significantly different (Mars et al., 2016).

#### 2.7.2 Evaluate individual patient fingerprints

Each patient’s task fingerprint at each visit was compared to the corresponding ‘normal’ task network, created by averaging the fingerprints of all controls (Voets et al., 2019). A given patient’s fingerprint was considered ‘atypical’ when its distance to the normal template was statistically greater than expected by chance (p < 0.05). Bonferroni (multiple comparisons) correction was applied to the single subject p-values.

#### 2.7.3 Quantify pre- and post-operative fingerprint alterations

This analysis followed the approach for single-subject comparisons. Here, the pre-operative task fingerprint for a given patient (rather than the control network) was used as the ‘template’ against which to test the post-operative task fingerprint for the same patient.

### 2.8 Accounting for potential tumour tissue confounds in connectivity analyses

The average pre-operative tumour volume was 39.1 cm^3^ (range 4.6–99.5 cm^3^). To explore potential confounding effects of tumour tissue within the anatomical masks used to generate the task fingerprints, we manually defined each patient’s tumour on their T1 weighted anatomical scan. We then generated a single mask, containing for each task separately all the seed and target masks. Next, we nonlinearly registered each patient’s tumour mask to the MNI standard atlas and computed the volume (mm^3^) of overlap between the tumour mask and the fingerprint masks. We then assigned patients to two groups: with and without overlap, and performed Chi-square tests comparing the incidence of atypical fluency or PPTT fingerprints according to presence (or not) of tumour tissue overlap with the network regions of interest.

### 2.9 Statistical analyses

Statistical analyses were performed using SPSS (v25) and Matlab (vR2018b). Nonparametric Mann-Whitney U and Wilcoxon rank-test were used for non-normally distributed data. Differences in atypical fingerprint incidence rates between categorical groups were assessed through Chi-square tests. Linear regression analyses were used to assess the influence of clinical variables, including tumour volume, histological grade, the presence of patient- or family-reported language symptoms at diagnosis, clinically-noted language impairment after surgery, and the time interval between surgery and the post-surgical scan. Significance was set at p < 0.05 (Bonferroni-corrected where reported).

### 2.10 Data availability

Fingerprint analysis scripts are freely available from www.neuroecologylab.org. Imaging data are available from the corresponding author upon reasonable request and ethical approval.

## 3 Results

### 3.1 Performance

**Fig. 2:**
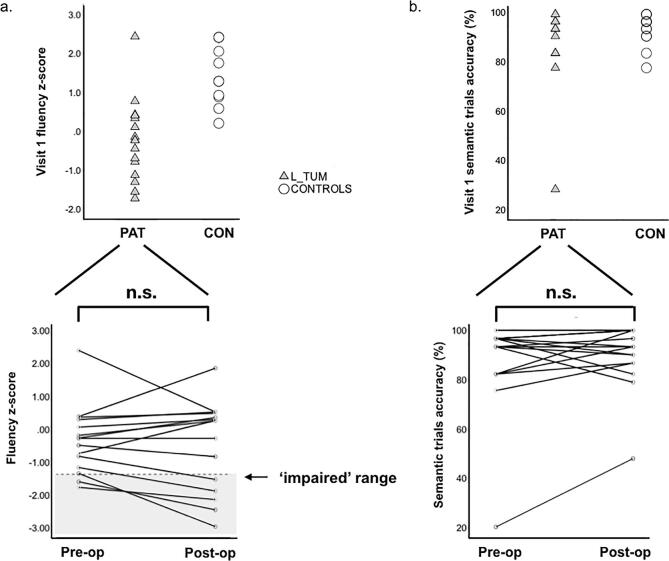
Phonemic fluency and PPTT performance before and after surgery *Legend*. Behavioural performance in brain tumour patients (PAT; L_TUM) and healthy controls (CON) for phonemic fluency (a) and the Pyramids and Palm Trees Test (PPTT) (b). The top row (scatter plots) depicts scores at the baseline visit (before surgery in patients). The lower row (spaghetti plots) track performance in individual patients before and after surgery. A z-score of −1.33 or below corresponds to a clinical impairment in verbal fluency, denoted by the light grey box. No standardised scores are currently available for the fMRI adaptation of the PPTT (which uses a subset of the full PPTT stimuli), therefore raw accuracy scores are reported. n.s. = non-significant (difference).

Before surgery fluency performance, assessing phonological language processing, was lower in patients than in controls (t = -5.3, p < 0.001) (Table 2). Performance was in the clinically impaired range (z-score ≤ −1.33) for 2 patients. After surgery, fluency was impaired in 5 patients; 2 patients retained their pre-operative impairment while 3 patients developed new deficits (Fig. 2a).

**Table 2:** Fluency and PPTT task performance.

	VISIT 1		VISIT 2	
Group	Fluency (z-score mean ± stdev (range))	PPTT (% accuracy, mean ± stdev (range))	Fluency (z-score mean ± stdev (range))	PPTT (accuracy %, mean ± stdev (range))
CON	1.53 ± 0.77 (0.2–2.41)	94.6 ± 5.9% (84–100)	N/A (same as V1)	98.4 ± 2.4% (97–100)
TUM	−0.25 ± 1.07 (−1.73–2.43)	89.5 ± 16.1% (55–100)	−0.50 ± 1.37 (−2.92–1.89)	91.5 ± 11.6% (64–100)
Sig.	*t = −5.28, p < 0.001*	*U = 124, p = 0.244*	*t = −5.02, p < 0.001*	*U = 80, p = 0.026*

*Legend*. Normalised fluency scores and raw percent accuracy on semantic trials of the fMRI Pyramids and Palm Trees Test (PPTT) in controls (CON) and tumour patients (TUM). The fMRI adaptation of the PPTT was generated from a sub-selection of the full neuropsychological PPTT task, hence normative (z-score) data are not available. Patients were assessed before (Visit 1) and after (Visit 2) surgery. Sig. = significance level. For nonparametric Mann-Whitney U-tests the exact significance is reported. Stdev = standard deviation.

PPTT accuracy, assessing semantic association performance, did not differ between patients and controls before surgery (U = 124, p = 0.24). Performance differed after surgery (U = 80, p = 0.026) (Fig. 2b, Table 2), due to improvements over time in controls (from 94.6% to 98.4%, Z = -2.84, p = 0.005). However, patient performance did not change pre- to post-surgery (Z = −0.92, p = 0.36).

### 3.2 Control fingerprints

To verify that semantic and phonological tasks engage expected spatially distinct brain regions, consistent with prevalent models of dissociated language processing (Poeppel et al., 2012), we first compared the whole-brain task connectivity maps between Por and Pop for both tasks in healthy controls. In controls, Por and Pop were functionally coupled with distinct networks of brain regions. Direct comparison of the whole-brain correlation maps revealed stronger coupling of fronto-parietal regions with Pop, and stronger coupling of temporal regions with Por, irrespective of task (Supplementary Fig. S1).

**Fig. 3:**
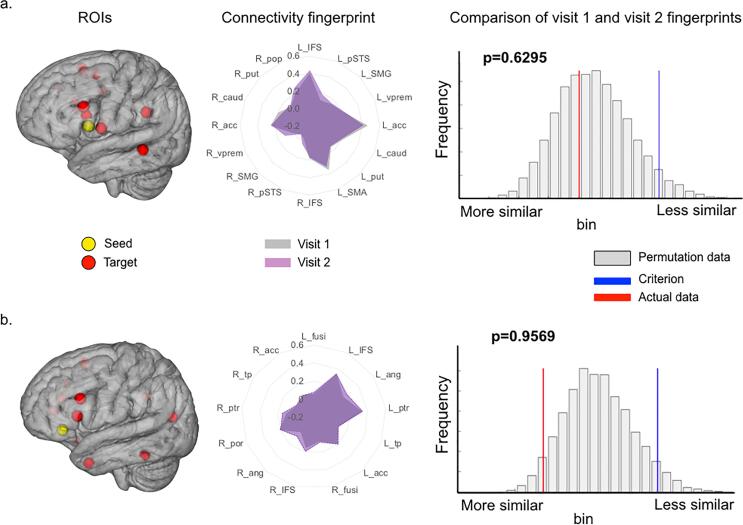
Language network fingerprint test–retest stability in controls *Legend.* Task-derived functional connectivity fingerprints in 17 healthy controls during performance of a word generation task (a), and a semantic association task (b). Fingerprints were measured on two visits, on average 4 months apart (mean 18 weeks, range: 4–37 weeks, chosen to match individual patient post-operative follow-up dates). For both tasks, the fingerprints of visit 1 and visit 2 were not significantly different (permutation testing, 10,000 permutations across all branches of the fingerprint and across all controls). The blue line (Criterion) shows the statistical threshold for significance on the permutation distribution while the red line shows the location of the actual data. To be significant (i.e. statistically ‘less similar’), the red lines would have needed to be to the right of the blue line (corresponding to p < 0.05). (For interpretation of the references to colour in this figure legend, the reader is referred to the web version of this article.)

Next, we generated functional connectivity ‘fingerprints’ representing phonological and semantic networks, separately, in each individual. The fingerprints of all healthy controls measured at their first visit were compared to the corresponding fingerprints re-measured on average 4.5 months later (matching the average post-surgical interval in patients). Both task fingerprints were highly reproducible in controls: permutation tests revealed no difference between the first and second scans for either fluency (p = 0.6295) or PPTT task fingerprints (p = 0.9569) (Fig. 3).

### 3.3 Pathological network disruption

Having found that the language networks remain stable under normal circumstances, we next evaluated firstly the baseline effect of brain tumours on each network among individual patients before any treatment, followed secondly by the effect of surgery on each network within individuals. For this analysis, each task fingerprint for each individual patient was tested against the ‘normal’ template network generated by averaging the fingerprints of healthy controls (separately for visit 1 and visit 2).

**Fig. 4:**
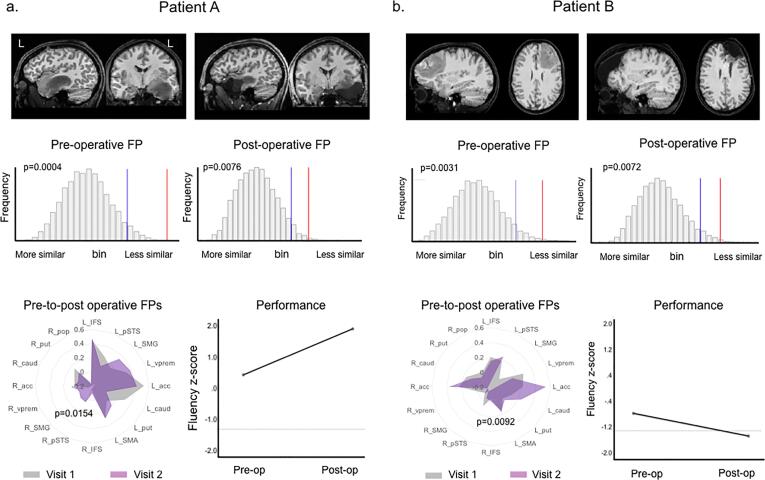
Fluency task fingerprints before and after tumour resection in 2 illustrative patients. *Legend*. Longitudinal comparison of fluency task fingerprints (FP) in 2 patients. In one patient with a left temporal lobe grade II astrocytoma (a), the pre-operative fluency network deviated significantly from the norm (middle row, permutation p-value = 0.0004). Five months after awake surgery, which resulted in a transient language deficit, the post-operative network remained atypical (comparison of the pre- (grey plot) and-post-operative (purple plot) FPs identified a statistical match (bottom row, p = 0.0154)). At this time, fluency performance had improved relative to pre-operative levels. In case b), with a diffuse left frontal grade II astrocytoma, the pre-operative fluency FP was likewise atypical compared to controls (though not significantly after Bonferroni correction (p = 0.0031), and remained atypical 6 months following surgery (direct comparison of pre- and post-operative FPs: p = 0.0092, indicating statistical match), but was accompanied by performance deterioration into the clinically impaired range. (For interpretation of the references to colour in this figure legend, the reader is referred to the web version of this article.)

Pre-operatively, the fluency fingerprint deviated significantly (Bonferroni-corrected results) from the typical network in 11/19 patients (57.9%, 7 ‘low grade’ and 4 ‘high grade’). For the semantic association task, 5/19 patient fingerprints (26.3%, 3 ‘low grade’ and 2 ‘high grade’) diverged from the normal network. Two examples showing diverging ways patient networks differed from the norm are shown in Fig. 4. The remaining patients are presented individually in Supplemental Figs. S3 and S4.

Importantly, the likelihood of either language network being atypical was not influenced simply by tumour tissue overlapping with the anatomical network masks (for fluency: χ (1) = 0.024, p = 0.87; for PPTT: χ (1) = 0.54, p = 0.46). Thus, atypical pre-operative language networks were not just the result of ‘no signal’ due to tumour within the network ROIs.

After surgery, 9/19 (47.4%, 3 ‘low grade’, 6 ‘high grade’) fluency-related fingerprints and 3/19 (15.8%, all ‘high grade’) PPTT-related fingerprints differed from the respective normal task-network. Again, no consistent pattern of reorganisation was seen (Fig. 4a vs 4b). When considering all results (pre-Bonferroni correction), the majority of patients with an atypical pre-operative network remained atypical after surgery (11/19 (57.9%) in the fluency task, 9/19 (47%) in PPTT). In 10% of patients, pre-operatively atypical networks reverted to a typical fingerprint post-operatively. Such ‘normalisation’ was apparent in 2 patients’ fluency fingerprints and in 2 (different) patients for PPTT. 3 of these patients had a grade II glioma and 1 patient a grade III glioma. Other patients developed newly atypical patterns only after surgery (2/19 (10.5%, both ‘high grade’) in the fluency task, 4/19 (21.1%, all ‘low grade’) in PPTT). Additionally, pre-operatively ‘normal’ networks could remain ‘typical’ when compared to controls, but nonetheless differ relative to the pre-operative network (2/4 fluency networks and 3/4 PPTT).

Both pre- and post-operatively, the fluency fingerprint could be atypical when the PPTT fingerprint was not, and post-surgery reorganisation could occur in one task network without the other. The likelihood of the global task fingerprint being atypical did not differ among the separate histological grades, or when patients were grouped into low-grade (WHO grade II) versus high grade (WHO grades III and IV) (all Chi-square test results p > 0.05). Results of repeating the PPTT analysis using the temporal pole as an alternative seed as presented in the Supplemental Results.

### 3.4 Clinical factors and language symptoms

Given the observed high inter-individual variability, we next explored clinical factors that might influence fingerprint distributions, by exploring relationships with the strength of connectivity in each part of the task networks among all patients. The presence of language symptoms at diagnosis, tumour histological grade and tumour volume correlated with pre-operative fluency fingerprint values, and together explained up to 51.8% in fingerprint connectivity. Among these, language symptoms at diagnosis was the strongest single predictor, accounting for 43.7% of fluency fingerprint variability. Only histological grade, tumour volume and age correlated with the pre-operative PPTT fingerprint, and explained up to 65% of fingerprint variance (Table 3).

**Table 3:** Impact of clinical variables on fingerprint variance.

PRE-OPERATIVE FINGERPRINT													
	Fluency							PPTT					
	Model			Predictor				Model			Predictor		
Region: Pop to	R^2^	F	p	Variable	β	p	Region: Por to	R^2^	F	P	Variable	β	p
L_vprem	0.44	3.9	0.031	Symptoms^†^	−0.62	0.010	L_fusi	0.29	3.4	0.044	Histology	−0.56	0.018
L_SMA	0.52	5.4	0.010	Histology	−0.49	0.021	L_ang	0.30	3.6	0.039	Age	−0.67	0.006
				Tumour volume	−0.54	0.017	R_ang	0.59	9.5	0.001	Histology	−0.58	0.003
							R_ang				Age	−0.67	0.001

CHANGE WITHIN PRE- TO POST-OPERATIVE FINGERPRINTS													
	Fluency							PPTT					
	Model			Predictor				Model			Predictor		
Region: Pop to	R^2^	F	p	Variable	β	p	Region: Por to	R^2^	F	P	Variable	β	p
L_pSTS	0.55	6.2	0.006	Deficit^‡^	0.65	0.003	L_ang	0.44	3.9	0.032	Histology	0.84	0.008
											Deficit^‡^	−0.5	0.033

*Legend*. Results of multiple linear regression analyses evaluating the contribution of clinical variables on task fingerprint connectivity. R^2^ denotes amount of variance explained by significant models (with corresponding F and p-values), followed by the beta weight(s) of the relevant clinical variable(s). † Language symptoms determined clinically at diagnosis. ‡ Clinically observed language deficits occurring immediately after surgery. L: left, R: right. Vprem: ventral premotor cortex. SMA: Supplementary motor area. pSTS: posterior superior temporal sulcus. Fusi: fusiform gyrus. Ang: angular gyrus.

Post-operatively, we evaluated whether histological grade, post-operative language symptoms, and scan interval from surgery (duration between surgery and post-operative MRI) contributed to a *change* in network connectivity (subtracting the post- from the pre-operative fingerprint). Only the presence of a post-operative language deficit contributed significantly to longitudinal fluency fingerprint changes, explaining up to 55% variance. In the semantic task, the presence of a post-operative deficit and histological grade together explained 43.5% of variance in PPTT fingerprint change (Table 3). The likelihood of a pre- to post-operative change in fingerprint connectivity did not differ according to scan interval (early pre-adjuvant treatment versus later post-operative scan)(fluency task: χ (1) = 0.046, p = 0.83, PPTT: χ (1) = 0.004, p = 0.95).

### 3.5 Performance association

These observed associations indicate a link between network configuration and symptom presence, which was based on limited categorical data: whether symptoms were clinically reported or not. We therefore performed an additional exploratory analysis to test if variations in the strength of functional connectivity within each network also correlated with task-specific performance across all patients. Treated as a group, fluency performance in tumour patients was correlated with functional connectivity between pars opercularis and the anterior cingulate pre-operatively (R = 0.55, p = 0.034), and with the inferior frontal sulcus region post-operatively (R = 0.61, p = 0.016). Furthermore, these preliminary data indicate that change in connectivity reflected change in fluency score at the group level (R = -0.54, p = 0.048) (Supplemental Fig. S2). Similarly, accuracy on the semantic trials correlated with functional connectivity between pars orbitalis and contralateral regions (angular gyrus pre-operatively, R = 0.57, p = 0.013; temporal pole post-operatively R = -0.59, p = 0.017). Mirroring the fluency task, change in functional connectivity to the right temporal pole was associated with a change in semantic trial accuracy, noting that semantic performance in general did not statistically decline after surgery (Supplemental Fig. S2). Given the number of branches in each network and the limited number of patients, these correlation results were not corrected for multiple comparisons.

## 4 Discussion

Deeper understanding of the mechanisms underlying brain network reorganisation has potential to improve clinical treatment planning across a wide range of neurological populations. In this longitudinal study, we measured the impact of one such treatment, brain tumour surgery, on complementary language networks at the single patient level. Using a recently-established connectivity fingerprinting method (Mars et al., 2013, Mars et al., 2016), we found that dominant-hemisphere surgery affects dorsal and ventral language systems independently of each other, and to different extents between patients. Notably, the presence of language symptoms, at diagnosis or after surgery, was the strongest predictor of altered network connectivity. Together, these results provide compelling support for a direct link between adaptive remodelling within brain networks and performance outcomes. Our approach, based on statistical comparison of individual network fingerprints, demonstrates translational potential to track patient-specific treatment-related neural adaptations within functional systems, of direct use to understand individual variability in treatment outcomes.

Reorganisation in language networks is well-established following acute injury such as stroke, chronic neurological conditions (Pillai, 2010) and gliomas (Cargnelutti et al., 2020). Interpretative challenges, however, arise from often widespread damage coupled with the absence of pre-injury data in these populations. In neurosurgical patients, pre-treatment data are routinely available, and the extent of surgical ‘damage’ is often well-controlled, especially in patients operated awake to monitor eloquent structures. Longitudinal case series and group-level studies show that task activation (Avramescu-Murphy et al., 2017) and connectivity (Deverdun et al., 2019) patterns are altered by surgery for tumours and epilepsy (Pillai, 2010). However, while some studies interpreted network changes as new inter- (Bonelli et al., 2012) or intra-hemispheric (Avramescu-Murphy et al., 2017) re-organisation, others observed normalisation (Chaudhary et al., 2017, Helmstaedter et al., 2006, Perrone-Bertolotti et al., 2012) or consolidation (Pataraia et al., 2005) of pre-operative activation patterns. Recent comprehensive reviews examine the evidence for post-surgical language-network plasticity and the potential contribution of left hemisphere *peri*-lesional versus contralateral structures in specific tasks (Cargnelutti et al., 2020, Duffau, 2020).

A fundamental limitation to understanding variability between patients arises from the typical reliance on group-level analyses. Group-level statistics aim to identify features that are *shared* among the study population. Consequently, in such analyses the very features which make individuals unique, and might account for wide variability in response to therapeutic interventions for example in stroke (Crinion and Leff, 2015), are lost. As one potential solution to this problem, here, we adopted a newly-developed approach based on single-subject connectivity fingerprinting (Mars et al., 2013, Mars et al., 2016) to track language network reorganisation after neurosurgery *within* patients. Mirroring high pre-operative inter-individual variability, we observed patient-unique adaptations in network connectivity after surgery. Approximately half the patients who had an atypical network before surgery remained atypical after surgery. A subset showed post-operative normalisation of pre-operatively atypical networks while others showed *de novo* re-organisation following surgery. The reasons for this inter-individual variability remain uncertain; the 4 patients whose networks normalised after surgery ranged in age (from 32 to 54), and histology (3 low-grade, 1 high grade, 2 oligodendroglioma, 2 astrocytoma, all IDH mutated). Furthermore, none of these tumours caused extensive *peri*-tumoral oedema such as often seen around a glioblastoma. Consequently, it does not appear that resolution of oedema alone explains normalisation of the functional networks in these patients. Nonetheless, these results help to reconcile divergence among previous group study results, and highlight that network adaptations do not follow a single set pattern. Instead, it appears essential to investigate how functional networks adapt at the individual level. Without being able to directly evaluate the functional importance of network adaptions after surgery, we cannot conclusively interpret our findings, but our results are at least in principle compatible with recent evidence in patients undergoing repeat awake surgery. Several studies have now found that brain regions that could not be resected during a first awake surgery can be removed at a second surgery, presumably because of functional adaptation (Duffau, 2014, Southwell et al., 2016). Of course direct brain stimulation can only assess the area directly in the site of surgery, and therefore the functionality of remote network regions identified with our fingerprinting method cannot be determined without further surgical data or additional methods such as transcranial magnetic stimulation. However, no previous studies have, to our knowledge, statistically quantified network-level reorganisation at the individual level pre- to post-surgery. Using this approach, we additionally observed that word-generation and semantic-association networks varied independently of each other. These tasks probe theoretically distinct but interacting ‘dorsal’ and ‘ventral’ language streams (Dick et al., 2014). Consequently, fingerprint analyses detect and track alterations at least partially specific to functional networks, and offer potential to begin differentiating indirect from behaviourally-useful network adaptations. A crucial consideration in the fingerprinting approach is of course the selection of regions to analyse. We explored the effect of varying the seed region in the PPTT analysis and demonstrate differences in both the categorisation of patients and the longitudinal effects of surgery on networks, according to how the network is defined. Therefore, the most appropriate set of regions may need to be tailored according either to the functions at greatest surgical risk, or according to the surgical approach intended in a given patient. Our demonstrated approach is certainly not intended to be considered perfect or exhaustive, and complementary assessment of structural connectivity will likely shed further light on brain parameters that may shape or limit network adaptation in individual patients.

The clinical and/or pathological factors that drive high inter-individual variability in network reorganisation remain uncertain. Tumour histopathological grade is thought to influence the potential for re-organisation (Duffau, 2015), due to implied differences in growth rate (faster in higher grades). Language re-organisation has, however, also been observed in high grade tumours (Briganti et al., 2012, Voets et al., 2019), and may therefore reflect a combination of tumour location and growth history/molecular status, as some high-grade tumours evolve from ‘low grade’ gliomas. In our series, histological grading influenced sub-components of each task fingerprint. However, the likelihood of the overall task fingerprint being atypical did not differ between tumour grades, although the small number of patients in each histological sub-group likely limits our sensitivity to detect such differences. We did not, for example, have a sufficient number of IDH wild-type patients (n = 4) to explore the effect of IDH status. Instead, the greatest contributor to network variance in our patient sample was the presence of language symptoms at diagnosis. These results indicate that among pathological processes, strain exerted on structures important for speech and language is among the strongest predictors of neural network re-configuration. This finding is in keeping with the concept that clinically relevant plasticity is ‘experience dependent’ (Cramer et al., 2011). In this framework, greater re-shaping of a given functional network would be expected when that network has been affected by a pathological process or intervention, as expressed by behavioural deficits eliciting adaptive change. The mere presence/absence of a language-related symptom is, of course, a subjective and non-specific metric. We did not have additional language test scores consistently available for all patients. However, further characterisation with objective tests is needed to replicate and better understand what type of behavioural changes influence network configuration in tumour patients.

Because of inevitable delays between diagnosis and scanning, we cannot differentiate if symptoms preceded network re-organisation or vice versa. Indeed, and crucially, the demonstration that language-related symptoms at onset or following surgery explained variance in aspects of fingerprint connectivity does not help to establish the causality of the relationship. It could be that the development of atypical networks contributes to the onset of pre-operative symptoms. It is interesting to note, however, that clinically impaired performance on the fluency task prior to surgery did not automatically translate into patients having a globally “atypical” network. Indeed, performance could be impaired while the fluency task overall remained ‘normal’, and vice versa: the fingerprint could be atypical while performance remained in the normal range. This finding supports evidence that not all patterns of network adaptation are behaviourally efficient (Cramer et al., 2011). Indeed, inefficient network reorganisation has been shown to contribute to memory deficits in epilepsy (Powell et al., 2007, Voets et al., 2014) and mild cognitive impairment (Bakker et al., 2012), while ‘aberrant’ network plasticity may sub-serve unique aspects of phantom limb conditions (Makin et al., 2015). Furthermore, since semantic performance was largely intact, further investigation in much larger groups showing a wide range of speech and language outcomes is needed to understand how re-allocations among network connections relate to specific aspects of language behaviour. We propose that connectivity fingerprinting, by offering a practical and easily interpretable metric to characterise functional networks in individual patients, offers promising future potential to differentiate patterns of network adaptation associated with efficient versus inefficient ‘plasticity’.

Specific challenges arise in longitudinal evaluation of functional networks at the single-patient level. Functional brain networks can be evaluated in many ways. The main issue for clinical application is how to determine in a sample size of 1 whether a network at time-point 1 differs from the network at time-point 2. By selecting network regions of interest from the literature, we attempted to implement an objective approach based on extensive prior research into language network organisation. However, the possibility remains that individual patients engage regions outside of these established regions. Different approaches, including whole-brain connectivity analyses could also be attempted, but raise similar questions in how functional brain parcels should be defined, alongside statistical challenges in reducing the output into a simple, clinically interpretable answer. Nonetheless, iterations on this approach certainly merit deeper investigation in larger cohorts, as our study has a modest sample size. Brain tumours are a rare condition, and although many additional patients were studied before surgery, there was a natural loss to post-operative follow-up of those patients with poor prognosis or very quick adjuvant treatment starts. A further limitation is that, for this initial demonstration, we acquired a single post-operative time-point. The role of individual brain regions has been shown to evolve between stages of impairment (Hartwigsen and Saur, 2017) or recovery (Saur et al., 2006). Therefore, additional scans would be informative to further characterise network adaptations occurring over time after surgery, although new confounds may arise in patients receiving adjuvant treatment. A third limitation is patient heterogeneity, including ‘high-’ and ‘low-’ grade pathology. Our aim was specifically to characterise network adaptation occurring within individual patients, irrespective of the type of glioma for which they were undergoing surgery. This is a short-coming when considered from the perspective of typical group-based analyses, but more closely reflects clinical practice, since tumour grade and type are not always radiologically certain before surgery. Clearly, larger studies focussing on individual tumour subtypes will be beneficial to further inform how pathological variations such as in molecular and genetic subtypes influence brain plasticity. Finally, it is not currently possible to differentiate network alterations occurring due to general cognitive upregulation (such as in effort, attention or working memory (Geranmayeh et al., 2014)), adoption of different strategies, or effective re-allocated functional roles. Assessing additional cognitive domains and validation in patients undergoing repeat awake surgery will be important to inform the clinical relevance of distinct network adaptation patterns.

## 5 Conclusions

In conclusion, our findings illustrate the importance of examining brain networks at the single patient level in order to gain insight into wide variations in symptoms and behavioural responses to pathology and to treatment among individuals. Our results build upon and extend previous work by demonstrating a practical approach to detect and monitor alterations in both global brain network patterns as well as specific brain network connections over time in individuals. In this way, connectivity fingerprinting provides a means to uncover specific adaptive mechanisms associated with good versus poor behavioural outcomes, stratify patients and monitor personalised treatment interventions. Potentially direct clinical applications include predicting patients at highest risk of treatment declines, timing surgery in line with network adaptation, and guiding neuromodulatory rehabilitative treatments aimed at specifically up-regulating behaviourally-beneficial connections.

## Declaration of Competing Interest

The authors declare that they have no known competing financial interests or personal relationships that could have appeared to influence the work reported in this paper.
