# Supplementary material for: Tracking longitudinal language network reorganisation using functional MRI connectivity fingerprints
Source: Neuroimage Clin. 2021 Apr 30;30:102689. doi: 10.1016/j.nicl.2021.102689 (PMC8122112; doi:10.1016/j.nicl.2021.102689)
Supplement: Supplementary data 1 [file mmc1.docx]

**Supplementary Methods**

*Comparison of whole-brain connectivity maps*

To compare functional connectivity maps from left Pars opercularis (Pop) and pars orbitalis (Por) during both language tasks, we performed permutations tests using FSL-randomise (Winkler, Ridgway, Webster, Smith, & Nichols, 2014). For this analysis, we used the whole-brain correlation maps to directly contrast the patterns of fMRI brain signal correlations between Por and Pop during the fluency and PPTT tasks, separately. This analysis confirmed findings reported in the literature indicating Por and Pop are preferentially engaged in dissociable language networks (Supplementary Fig S3).

**Supplemental Results**

*Effect of varying seed ROI in the PPTT fingerprint analysis*

To examine the effect of using different seed regions on the fingerprint analysis results, the PPTT network fingerprints were re-generated using the left temporal pole as seed region instead of left pars opercularis. Pars opercularis was instead used as a target region along-side the other same target regions as in the original analysis. Using the temporal pole as seed, pre-operatively, only 1 patient’s fingerprint statistically deviated from the respective healthy control network. Another 5 patients showed tendencies for an atypical network that did not survive statistical significance after correction for multiple comparisons. 4 of these patients were the same as originally identified as tending to ‘atypical’ in the original analysis using pars orbitalis as seed. Therefore, overall, 1 / 19 patients showed a different result when varying the seed. This patient had a left frontal lobe grade II glioma; their PPTT fingerprint was deemed typical when using pars orbitalis as a seed, but atypical when seeding from the temporal pole.

Post-operatively, 5 patients showed exactly the same pattern of results with the temporal pole seed as originally observed using the pars orbitalis seed. Of these 5, 2 had a pre-operatively typical fingerprint that stayed typical; 2 had a pre-operatively atypical network that stayed atypical, and 1 had a pre-operatively typical network that became atypical after surgery. The remaining patients showed a range of pre-to-post-operative network changes: 6 remained typical, 4 patients became atypical, and 4 pre-operatively atypical became typical after surgery.

**Supplementary Table 1. FMRI sequence acquisition parameters.**

| **Task** | **Siemens Verio (3 controls, 7 patients)** | **Siemens Prisma (14 controls, 13 patients)** |
| --- | --- | --- |
| Letter fluency | TR = 3000ms, TE = 28ms, voxel size = 3mm^2^, GRAPPA acceleration factor 2, FOV = 192mm^2^, matrix size = 64, 44 slices, duration 04:12mins. | TR = 933ms, TE = 33.4ms, voxel size = 2mm^2^, multiband acceleration factor = 6, FOV = 192mm^2^, matrix size = 96, 72 slices, duration 04:18mins. |
| Pyramids and Palm Trees Test | TR = 3000ms, TE = 28ms, voxel size = 3mm^2^, GRAPPA acceleration factor 2, FOV = 192mm^2^, matrix size = 64, 44 slices, duration 05:27mins. | TR = 933ms, TE = 33.4ms, voxel size = 2mm^2^, multiband acceleration factor = 6, FOV = 192mm^2^, matrix size = 96, 72 slices, duration 05:36mins. |

***Legend***. Data were acquired on 3T Siemens MRI systems and the University of Oxford Wellcome Centre for Integrative Neuroimaging (WIN-FMRIB Centre). Participants were scanned on a ‘Verio’ or a ‘Prisma’ system, due to a scanner replacement during the study period. Longitudinal data were acquired on the same system for all healthy controls, and for 17 of 20 patients. Three patients, pre-operatively assessed on the Verio scanner, were re-evaluated post-operatively on the Prisma system. We have previously demonstrated that network fingerprints for the fluency task were equivalent between these two scanners (Voets et al., 2019).

**Supplementary Table 2. Anatomical mask coordinates for the ‘word generation’ (fluency) network**.

| Region | Hemisphere | MNI Coordinates (mm) | | |
| --- | --- | --- | --- | --- |
|  |  | x | y | z |
| Ventral pars opercularis | L | -52 | 16 | 8 |
|  | R | 54 | 18 | 8 |
| Inferior frontal sulcus | L | -51 | 25 | 25 |
|  | R | 52 | 26 | 22 |
| Dorsal anterior cingulate gyrus | L | -6 | 20 | 34 |
|  | R | 8 | 20 | 36 |
| Posterior superior temporal sulcus | L | -66 | -34 | -1 |
|  | R | 66 | -34 | -2 |
| Caudate nucleus | L | -12 | 8 | 10 |
|  | R | 14 | 10 | 10 |
| Putamen | L | -24 | -6 | 6 |
|  | R | 24 | -4 | 6 |
| Supramarginal gyrus | L | -52 | -34 | 30 |
|  | R | 54 | -24 | 28 |
| Ventral premotor cortex | L | -52 | 4 | 8 |
|  | R | 54 | 4 | 10 |
| Pre-supplementary motor area | L | -4 | 7 | 50 |

*Legend*. Connectivity fingerprints were generated by calculating signal correlation during performance of a covert phonemic fluency functional MRI task between the left ventral pars opercularis and 16 target regions forming part of the wider speech-related language network. A 5mm spherical region-of-interest mask was created for each region, centred on x, y, z mm coordinates selected from the literature. Coordinates are reported in Montreal Neurological Institute (MNI) space.

**Supplementary Table 3. Anatomical mask coordinates used to generate the ‘semantic association’ (Pyramids and Palm Trees Task) network.**

| Region | Hemisphere | MNI Coordinates (mm) | | |
| --- | --- | --- | --- | --- |
|  |  | x | y | z |
| Pars orbitalis | L | -45 | 34 | -8 |
|  | R | 45 | 34 | 8 |
| Fusiform gyrus | L | -46 | -46 | -20 |
|  | R | 44 | -44 | -20 |
| Inferior frontal sulcus | L | -51 | 25 | 25 |
|  | R | 52 | 26 | 22 |
| Angular gyrus | L | -48 | -64 | 20 |
|  | R | 48 | -64 | 22 |
| Pars triangularis | L | -50 | 24 | 8 |
|  | R | 48 | 28 | 6 |
| Temporal pole | L | -42 | 4 | -36 |
|  | R | 42 | 6 | -36 |
| Dorsal anterior cingulate gyrus | L | -6 | 20 | 34 |
|  | R | 8 | 20 | 36 |

*Legend.* Functional connectivity fingerprints were generated by calculating signal correlation during performance of a visual semantic association functional MRI task between the left ventral pars orbitalis and 13 target regions forming part of the distributed semantic language network. A 5mm spherical region-of-interest mask was created for each region, centred on x, y, z mm coordinates selected from the literature. Coordinates are reported in Montreal Neurological Institute (MNI) space.

**Supplementary Fig S1. Task-based whole brain functional connectivity maps.**


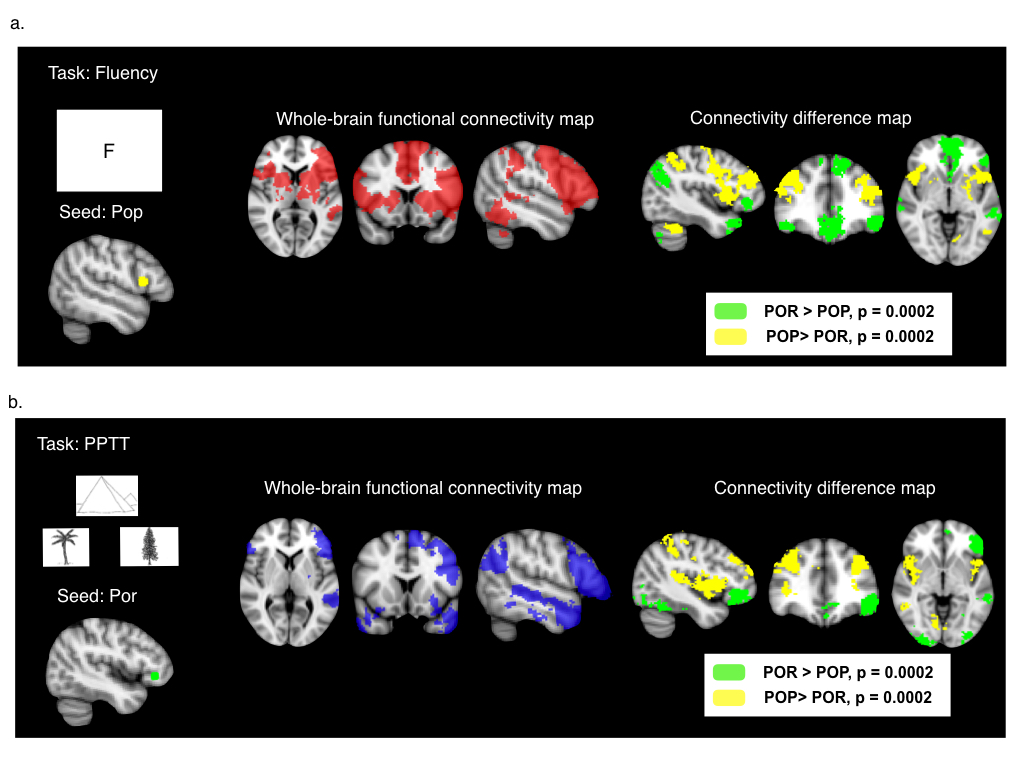


*Legend*. Seed-based correlation maps between seed regions in the inferior frontal gyrus and all voxels of the brain during performance of a word generation task (a) and a semantic matching task (b) in healthy controls. FMRI signal correlation between pars opercularis (Pop) and the rest of the brain during the fluency task identified a characteristic network of brain regions involved in expressive language functions. In comparison, FMRI signal correlation between pars orbitalis (Por) and the rest of the brain during the Pyramids and Palm Trees Task (PPTT) highlighted brain regions commonly attributed to the semantic processing system. Direct comparison of the whole-brain correlation maps derived from Pop and Por identified differential connectivity patterns of these inferior frontal sub-regions (greater connectivity of Por with the semantic network and preferential connectivity of Pop with the word generation network), irrespective of task (p<0.001).

**Supplementary Fig S2. Correlations between task-based performance and functional connectivity within each network.**

**
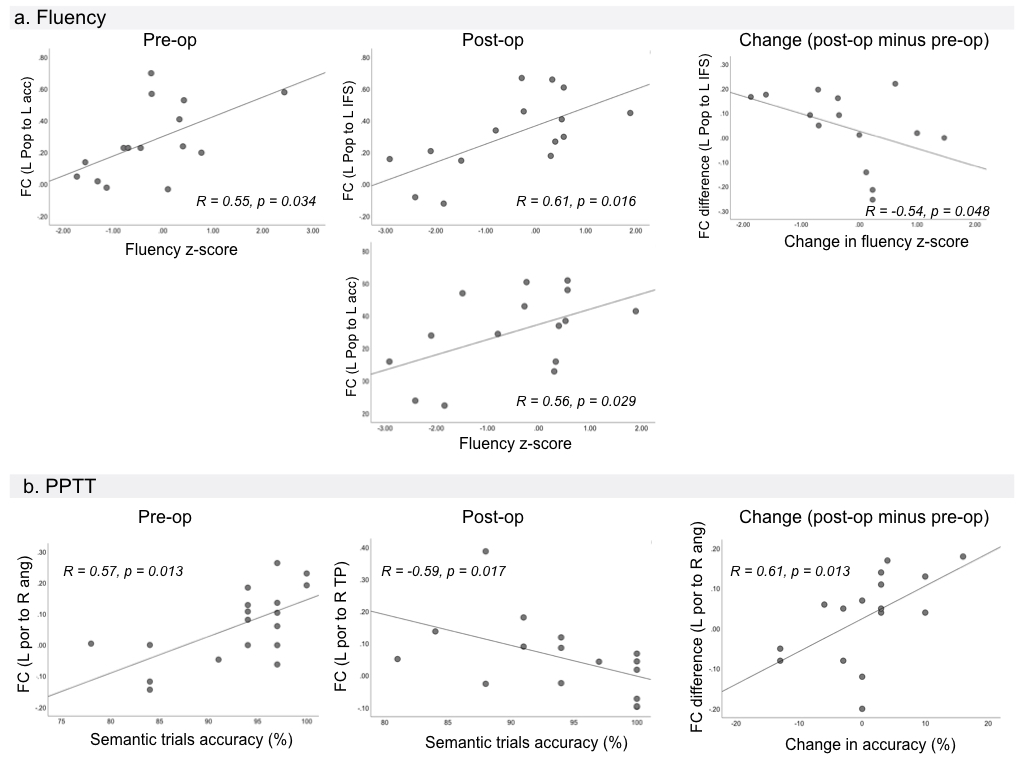
**

*Legend*. Spearman correlations assessing relationships between task performance and functional connectivity (FC) of each of the network fingerprint branches. a) Fluency task performance correlated with FC strength between pars opercularis (Pop) and branches of the task network before and after surgery, particularly the left anterior cingulate (ACC) and inferior frontal sulcus (IFS). A larger post-operative change in FC with the left IFS was associated with a decline in performance. b) Similarly, performance on the pyramids and palm trees task (PPTT) was associated with FC in branches of the task network, especially the contralateral right angular gyrus (ang) and temporal pole (TP). Post-operative change in FC was associated with a change in accuracy: greater recruitment of the right TP was associated with a larger change in semantic trial accuracy, noting that semantic performance did not statistically decline. The PPTT results are presented after excluding one outlier who performed very poorly; the trends are the same with or without this patient. Correlation values are reported uncorrected for multiple comparisons.

**Supplementary Fig S3. Pre- and post-operative fluency fingerprints in all patients**

**
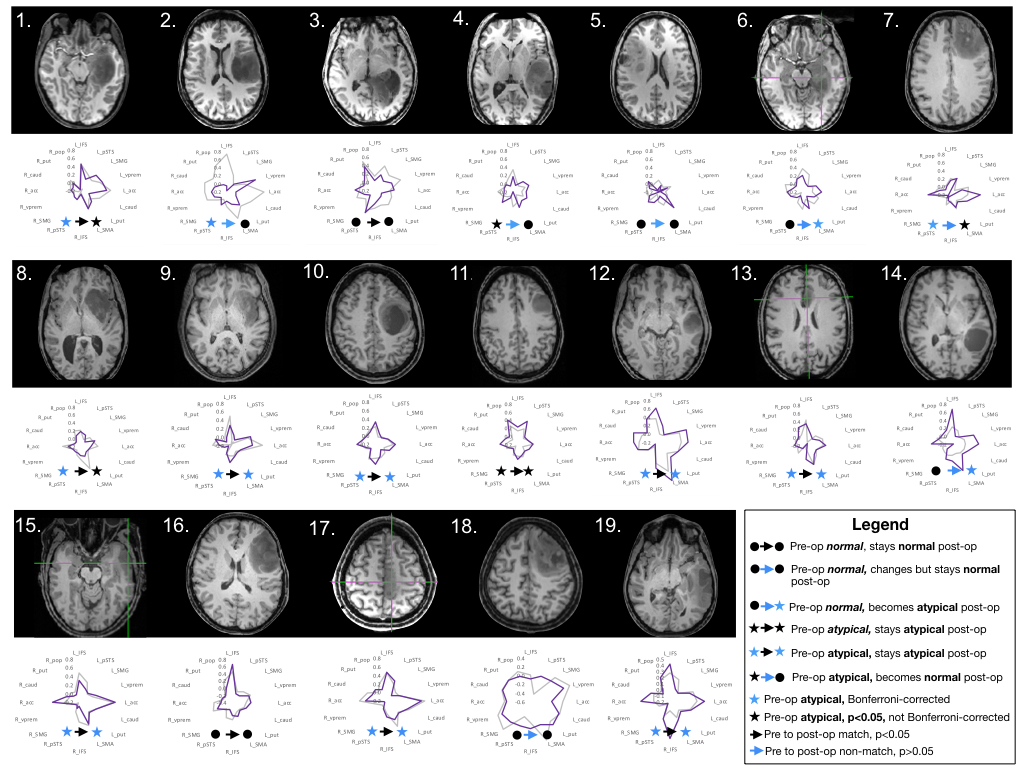
**

*Legend.* Pre-operative T1 structural scan of the tumour in each of the 19 patients. Note patient 5 had a tumour in the right frontal lobe, but was left-handed and had atypical right-hemisphere dominance for language. Corresponding connectivity fingerprints representing the fluency task-related language network before surgery (grey line) and after surgery (purple line) in each patient. Pre-operative fingerprints were statistically compared to a template network derived from healthy controls to determine if individual patient fingerprints were ‘typical’ or ‘atypical’. Post-operative fingerprints were statistically compared to the respective pre-operative fingerprint to identify post-surgery adaptation (statistical deviation from the pre-operative network or not).

**Supplementary Fig S4. Pre- and post-operative PPTT fingerprints in all patients**

**
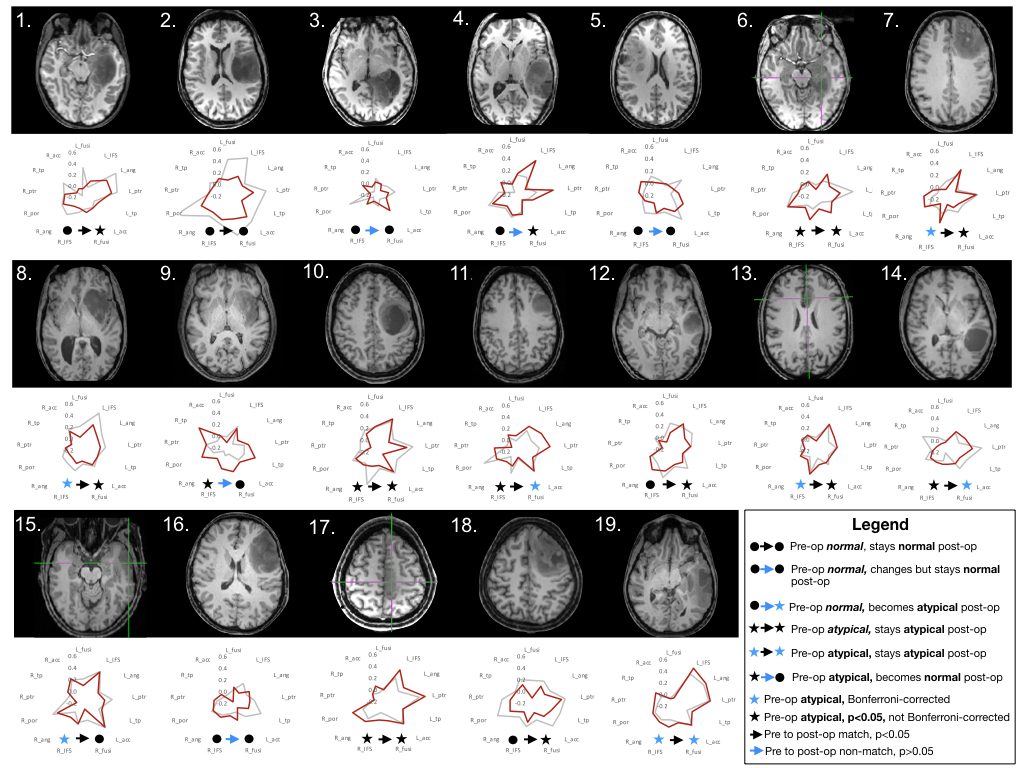
**

*Legend*. Pre-operative T1 structural scan of the tumour in each of the 19 patients and corresponding connectivity fingerprints representing the semantic association (Pyramids and Palm Trees Test) language network before surgery (grey line) and after surgery (red line) in each patient. Pre-operative fingerprints were statistically compared to a template network derived from healthy controls to determine if individual patient fingerprints were ‘typical’ or ‘atypical’. Post-operative fingerprints were statistically compared to the respective pre-operative fingerprint to identify post-surgery adaptation (statistical deviation from the pre-operative network or not).

**Supplemental Figure S5. Overlap map of the surgical resection areas.**


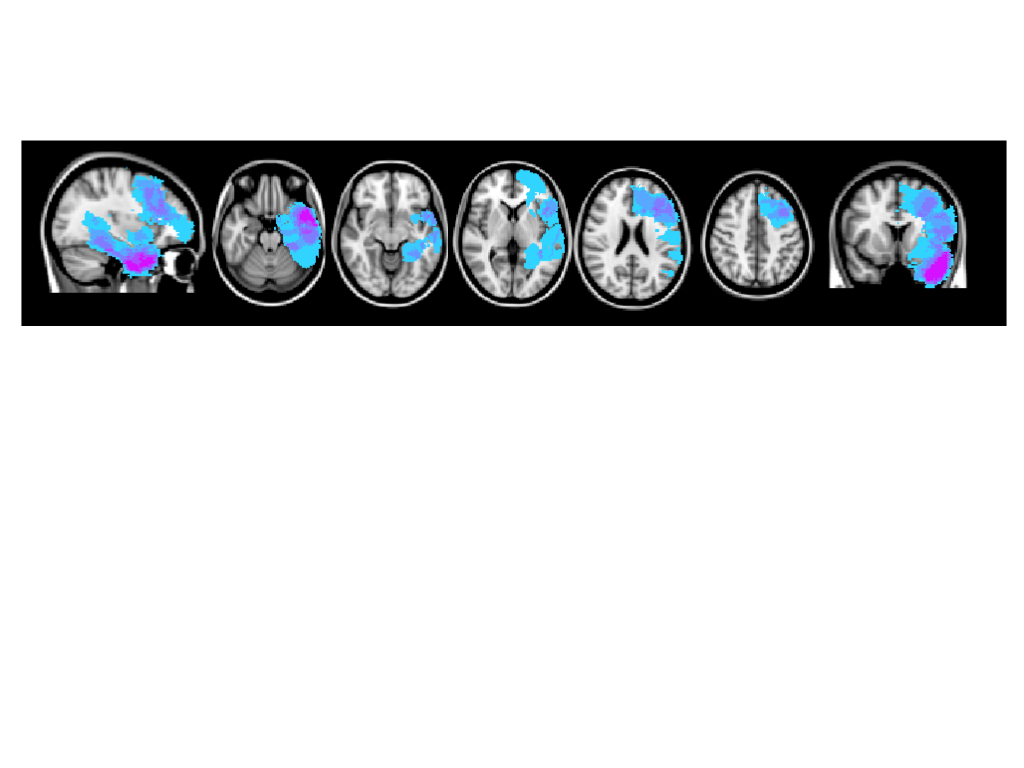


*Legend*. Overlap map indicating the areas of tumour resection, generated from manual masks of the resection cavities in the 19 patients.

**Supplementary References**

Voets, N. L., Parker Jones, O., Mars, R. B., Adcock, J. E., Stacey, R., Apostolopoulos, V., & Plaha, P. (2019). Characterising neural plasticity at the single patient level using connectivity fingerprints. *Neuroimage Clin, 24*, 101952. doi:10.1016/j.nicl.2019.101952

Winkler, A. M., Ridgway, G. R., Webster, M. A., Smith, S. M., & Nichols, T. E. (2014). Permutation inference for the general linear model. *Neuroimage, 92*, 381-397. doi:10.1016/j.neuroimage.2014.01.060
